# Supplementary material for: Modeling glioblastoma heterogeneity as a dynamic network of cell states
Source: Mol Syst Biol. 2021 Sep 16;17(9):e10105. doi: 10.15252/msb.202010105 (PMC8444284; doi:10.15252/msb.202010105)
Supplement: Supplementary file 5 — Source Data for Figure 3 [file MSB-17-e10105-s001.zip › Figure3A_sourcedata/GSEA_3065/nosigdb_state5.GseaPreranked.1622718435914/FELDMAN_2020_G2-M.html]

Details for gene set FELDMAN\_2020\_G2-M[GSEA]

|  || Dataset | state5 |
| Phenotype | NoPhenotypeAvailable |
| Upregulated in class | na\_neg |
| GeneSet | FELDMAN\_2020\_G2-M |
| Enrichment Score (ES) | -0.49626952 |
| Normalized Enrichment Score (NES) | -1.8878791 |
| Nominal p-value | 0.0 |
| FDR q-value | 5.350027E-4 |
| FWER p-Value | 0.011 |
Table: GSEA Results Summary

  

Fig 1: Enrichment plot: FELDMAN\_2020\_G2-M      
 Profile of the Running ES Score & Positions of GeneSet Members on the Rank Ordered List

  

| PROBE | GENE SYMBOL | GENE\_TITLE | RANK IN GENE LIST | RANK METRIC SCORE | RUNNING ES | CORE ENRICHMENT || 1 | MALAT1 |  |  | 0 | 1.196 | 0.0158 | No |
| 2 | NOVA1 |  |  | 10 | 0.870 | 0.0263 | No |
| 3 | PTPRZ1 |  |  | 12 | 0.862 | 0.0376 | No |
| 4 | PTN |  |  | 19 | 0.824 | 0.0478 | No |
| 5 | SCRG1 |  |  | 36 | 0.715 | 0.0554 | No |
| 6 | GPM6B |  |  | 41 | 0.702 | 0.0643 | No |
| 7 | TNC |  |  | 45 | 0.680 | 0.0729 | No |
| 8 | MIAT |  |  | 58 | 0.619 | 0.0797 | No |
| 9 | FGFBP3 |  |  | 85 | 0.545 | 0.0839 | No |
| 10 | PRCP |  |  | 103 | 0.523 | 0.0889 | No |
| 11 | ITM2B |  |  | 117 | 0.509 | 0.0941 | No |
| 12 | TTYH1 |  |  | 141 | 0.489 | 0.0980 | No |
| 13 | CLU |  |  | 162 | 0.474 | 0.1020 | No |
| 14 | CNIH4 |  |  | 169 | 0.465 | 0.1074 | No |
| 15 | GGH |  |  | 184 | 0.453 | 0.1118 | No |
| 16 | LBR |  |  | 198 | 0.440 | 0.1161 | No |
| 17 | CTGF |  |  | 199 | 0.440 | 0.1220 | No |
| 18 | PSAP |  |  | 227 | 0.420 | 0.1244 | No |
| 19 | ARL6IP1 |  |  | 232 | 0.418 | 0.1295 | No |
| 20 | PIF1 |  |  | 266 | 0.400 | 0.1310 | No |
| 21 | SPARC |  |  | 327 | 0.370 | 0.1290 | No |
| 22 | TM7SF2 |  |  | 352 | 0.360 | 0.1310 | No |
| 23 | IGFBP7 |  |  | 424 | 0.333 | 0.1273 | No |
| 24 | TXNDC12 |  |  | 527 | 0.304 | 0.1195 | No |
| 25 | HES6 |  |  | 653 | 0.274 | 0.1088 | No |
| 26 | DCBLD2 |  |  | 863 | 0.230 | 0.0878 | No |
| 27 | HSD17B11 |  |  | 949 | 0.219 | 0.0810 | No |
| 28 | ASPM |  |  | 980 | 0.213 | 0.0803 | No |
| 29 | PLP1 |  |  | 1022 | 0.208 | 0.0784 | No |
| 30 | EIF4A2 |  |  | 1153 | 0.191 | 0.0659 | No |
| 31 | PCSK1N |  |  | 1162 | 0.190 | 0.0675 | No |
| 32 | SERPINE1 |  |  | 1221 | 0.182 | 0.0633 | No |
| 33 | MPHOSPH9 |  |  | 1256 | 0.178 | 0.0617 | No |
| 34 | IFITM3 |  |  | 1332 | 0.168 | 0.0553 | No |
| 35 | RBMX |  |  | 1512 | 0.151 | 0.0368 | No |
| 36 | CENPF |  |  | 1531 | 0.149 | 0.0367 | No |
| 37 | PLD3 |  |  | 1562 | 0.145 | 0.0351 | No |
| 38 | CST3 |  |  | 1602 | 0.141 | 0.0325 | No |
| 39 | GPSM2 |  |  | 1612 | 0.141 | 0.0333 | No |
| 40 | MIS18BP1 |  |  | 1765 | 0.129 | 0.0176 | No |
| 41 | REEP4 |  |  | 1793 | 0.127 | 0.0162 | No |
| 42 | HNRNPA2B1 |  |  | 1852 | 0.122 | 0.0111 | No |
| 43 | KIF20B |  |  | 1962 | 0.114 | 0.0001 | No |
| 44 | IGFBP3 |  |  | 2037 | 0.109 | -0.0070 | No |
| 45 | MEST |  |  | 2059 | 0.108 | -0.0080 | No |
| 46 | MXD3 |  |  | 2178 | 0.100 | -0.0202 | No |
| 47 | GLIPR1 |  |  | 2261 | 0.094 | -0.0284 | No |
| 48 | DIAPH3 |  |  | 2387 | 0.086 | -0.0416 | No |
| 49 | MELK |  |  | 2402 | 0.085 | -0.0421 | No |
| 50 | SFPQ |  |  | 2548 | 0.077 | -0.0578 | No |
| 51 | RNASEH2A |  |  | 2560 | 0.076 | -0.0580 | No |
| 52 | TMEM237 |  |  | 2680 | 0.069 | -0.0708 | No |
| 53 | CCDC18 |  |  | 2814 | 0.062 | -0.0852 | No |
| 54 | PRPSAP1 |  |  | 2822 | 0.061 | -0.0852 | No |
| 55 | AURKB |  |  | 2853 | 0.060 | -0.0879 | No |
| 56 | RTKN2 |  |  | 2869 | 0.059 | -0.0888 | No |
| 57 | CKAP2L |  |  | 2979 | 0.053 | -0.1007 | No |
| 58 | HP1BP3 |  |  | 3122 | 0.046 | -0.1164 | No |
| 59 | NCAPG |  |  | 3194 | 0.041 | -0.1240 | No |
| 60 | IGFBP5 |  |  | 3230 | 0.040 | -0.1275 | No |
| 61 | FOXM1 |  |  | 3279 | 0.038 | -0.1325 | No |
| 62 | BUB1B |  |  | 3355 | 0.034 | -0.1407 | No |
| 63 | SMC2 |  |  | 3374 | 0.032 | -0.1423 | No |
| 64 | G2E3 |  |  | 3493 | 0.028 | -0.1555 | No |
| 65 | DEPDC1B |  |  | 3555 | 0.025 | -0.1622 | No |
| 66 | HOXB4 |  |  | 3600 | 0.023 | -0.1670 | No |
| 67 | KIFC1 |  |  | 3667 | 0.021 | -0.1743 | No |
| 68 | ESCO2 |  |  | 3672 | 0.021 | -0.1745 | No |
| 69 | GAS2L3 |  |  | 3714 | 0.020 | -0.1789 | No |
| 70 | SPC24 |  |  | 3726 | 0.019 | -0.1799 | No |
| 71 | GTSE1 |  |  | 3738 | 0.018 | -0.1809 | No |
| 72 | PARPBP |  |  | 3820 | 0.015 | -0.1900 | No |
| 73 | DLGAP5 |  |  | 3907 | 0.011 | -0.1998 | No |
| 74 | NCAPD2 |  |  | 3914 | 0.011 | -0.2003 | No |
| 75 | NDC80 |  |  | 4034 | 0.006 | -0.2139 | No |
| 76 | TNFAIP8L1 |  |  | 4236 | -0.003 | -0.2370 | No |
| 77 | PRC1 |  |  | 4241 | -0.003 | -0.2374 | No |
| 78 | EMC9 |  |  | 4405 | -0.010 | -0.2560 | No |
| 79 | TACC3 |  |  | 4450 | -0.012 | -0.2609 | No |
| 80 | HJURP |  |  | 4497 | -0.014 | -0.2660 | No |
| 81 | CKAP5 |  |  | 4519 | -0.015 | -0.2682 | No |
| 82 | DCP2 |  |  | 4575 | -0.017 | -0.2743 | No |
| 83 | KIF2C |  |  | 4621 | -0.019 | -0.2793 | No |
| 84 | NUF2 |  |  | 4628 | -0.019 | -0.2797 | No |
| 85 | CENPK |  |  | 4675 | -0.021 | -0.2847 | No |
| 86 | CALD1 |  |  | 4714 | -0.022 | -0.2888 | No |
| 87 | NUP35 |  |  | 4749 | -0.024 | -0.2924 | No |
| 88 | KIF14 |  |  | 4758 | -0.024 | -0.2930 | No |
| 89 | CDK1 |  |  | 4766 | -0.025 | -0.2935 | No |
| 90 | CENPE |  |  | 4787 | -0.025 | -0.2954 | No |
| 91 | PRR11 |  |  | 4822 | -0.026 | -0.2990 | No |
| 92 | HMMR |  |  | 4826 | -0.027 | -0.2990 | No |
| 93 | SHCBP1 |  |  | 4848 | -0.027 | -0.3010 | No |
| 94 | TROAP |  |  | 4869 | -0.028 | -0.3030 | No |
| 95 | DDX39A |  |  | 4948 | -0.031 | -0.3115 | No |
| 96 | KIF18A |  |  | 4962 | -0.032 | -0.3126 | No |
| 97 | BTG1 |  |  | 5087 | -0.037 | -0.3264 | No |
| 98 | FILIP1L |  |  | 5096 | -0.037 | -0.3268 | No |
| 99 | CCNB1 |  |  | 5111 | -0.037 | -0.3279 | No |
| 100 | PSRC1 |  |  | 5137 | -0.038 | -0.3303 | No |
| 101 | BUB1 |  |  | 5195 | -0.040 | -0.3363 | No |
| 102 | SPAG5 |  |  | 5225 | -0.041 | -0.3391 | No |
| 103 | ECT2 |  |  | 5264 | -0.043 | -0.3429 | No |
| 104 | SPC25 |  |  | 5312 | -0.045 | -0.3477 | No |
| 105 | CCNB2 |  |  | 5382 | -0.048 | -0.3550 | No |
| 106 | NUSAP1 |  |  | 5389 | -0.048 | -0.3550 | No |
| 107 | NEK2 |  |  | 5442 | -0.050 | -0.3604 | No |
| 108 | SPDL1 |  |  | 5460 | -0.051 | -0.3616 | No |
| 109 | FAM83D |  |  | 5467 | -0.051 | -0.3617 | No |
| 110 | TRIP13 |  |  | 5570 | -0.056 | -0.3726 | No |
| 111 | BUB3 |  |  | 5596 | -0.057 | -0.3748 | No |
| 112 | TTK |  |  | 5604 | -0.058 | -0.3748 | No |
| 113 | RNF26 |  |  | 5657 | -0.060 | -0.3800 | No |
| 114 | CCNA2 |  |  | 5671 | -0.061 | -0.3807 | No |
| 115 | TOP2A |  |  | 5722 | -0.063 | -0.3856 | No |
| 116 | CDCA3 |  |  | 5726 | -0.063 | -0.3851 | No |
| 117 | KIF22 |  |  | 5758 | -0.064 | -0.3878 | No |
| 118 | KIF11 |  |  | 5795 | -0.065 | -0.3911 | No |
| 119 | KPNA2 |  |  | 5811 | -0.066 | -0.3920 | No |
| 120 | CDCA8 |  |  | 5863 | -0.068 | -0.3969 | No |
| 121 | RACGAP1 |  |  | 5902 | -0.070 | -0.4004 | No |
| 122 | KNSTRN |  |  | 5923 | -0.070 | -0.4017 | No |
| 123 | SHISA4 |  |  | 5957 | -0.072 | -0.4046 | No |
| 124 | EXOSC8 |  |  | 6020 | -0.075 | -0.4107 | No |
| 125 | FOSL1 |  |  | 6131 | -0.082 | -0.4223 | No |
| 126 | ATP6V0E1 |  |  | 6138 | -0.082 | -0.4219 | No |
| 127 | LRR1 |  |  | 6149 | -0.082 | -0.4219 | No |
| 128 | MKI67 |  |  | 6211 | -0.086 | -0.4278 | No |
| 129 | RRM2 |  |  | 6308 | -0.091 | -0.4376 | No |
| 130 | LIMA1 |  |  | 6339 | -0.092 | -0.4399 | No |
| 131 | CENPM |  |  | 6368 | -0.093 | -0.4419 | No |
| 132 | RUVBL2 |  |  | 6371 | -0.093 | -0.4409 | No |
| 133 | CKAP2 |  |  | 6372 | -0.094 | -0.4396 | No |
| 134 | CENPA |  |  | 6374 | -0.094 | -0.4385 | No |
| 135 | POC1A |  |  | 6411 | -0.096 | -0.4414 | No |
| 136 | ARHGAP11A |  |  | 6423 | -0.097 | -0.4413 | No |
| 137 | ISCA2 |  |  | 6449 | -0.098 | -0.4429 | No |
| 138 | ZWINT |  |  | 6453 | -0.099 | -0.4419 | No |
| 139 | SMC4 |  |  | 6517 | -0.102 | -0.4478 | No |
| 140 | KIF4A |  |  | 6529 | -0.103 | -0.4477 | No |
| 141 | DBF4 |  |  | 6572 | -0.106 | -0.4511 | No |
| 142 | PLK1 |  |  | 6581 | -0.107 | -0.4507 | No |
| 143 | MAD2L1 |  |  | 6600 | -0.108 | -0.4513 | No |
| 144 | TP53I3 |  |  | 6607 | -0.108 | -0.4505 | No |
| 145 | HNRNPDL |  |  | 6625 | -0.109 | -0.4511 | No |
| 146 | LMNB1 |  |  | 6638 | -0.110 | -0.4510 | No |
| 147 | ANP32E |  |  | 6655 | -0.111 | -0.4513 | No |
| 148 | CEP55 |  |  | 6700 | -0.114 | -0.4549 | No |
| 149 | DEPDC1 |  |  | 6723 | -0.115 | -0.4559 | No |
| 150 | CDCA2 |  |  | 6730 | -0.116 | -0.4551 | No |
| 151 | RHNO1 |  |  | 6734 | -0.116 | -0.4539 | No |
| 152 | CBX3 |  |  | 6756 | -0.118 | -0.4547 | No |
| 153 | GOT1 |  |  | 6759 | -0.118 | -0.4534 | No |
| 154 | KIF23 |  |  | 6764 | -0.118 | -0.4523 | No |
| 155 | FBXO5 |  |  | 6775 | -0.119 | -0.4519 | No |
| 156 | CCDC34 |  |  | 6826 | -0.123 | -0.4560 | No |
| 157 | ANLN |  |  | 6829 | -0.123 | -0.4546 | No |
| 158 | BCL2L12 |  |  | 6853 | -0.125 | -0.4556 | No |
| 159 | HSP90AA1 |  |  | 6962 | -0.134 | -0.4662 | No |
| 160 | DDIT4 |  |  | 6968 | -0.135 | -0.4650 | No |
| 161 | UBC |  |  | 6969 | -0.135 | -0.4632 | No |
| 162 | CDC20 |  |  | 6985 | -0.136 | -0.4632 | No |
| 163 | AURKA |  |  | 6990 | -0.136 | -0.4618 | No |
| 164 | PBK |  |  | 7010 | -0.138 | -0.4622 | No |
| 165 | CDC27 |  |  | 7083 | -0.144 | -0.4685 | No |
| 166 | CDC25B |  |  | 7102 | -0.146 | -0.4687 | No |
| 167 | C4orf48 |  |  | 7110 | -0.146 | -0.4676 | No |
| 168 | MIS18A |  |  | 7115 | -0.147 | -0.4661 | No |
| 169 | CENPW |  |  | 7116 | -0.147 | -0.4641 | No |
| 170 | GMNN |  |  | 7224 | -0.158 | -0.4743 | No |
| 171 | TMPO |  |  | 7296 | -0.167 | -0.4803 | No |
| 172 | CENPH |  |  | 7329 | -0.171 | -0.4817 | No |
| 173 | HNRNPH3 |  |  | 7361 | -0.176 | -0.4830 | No |
| 174 | TK1 |  |  | 7377 | -0.178 | -0.4823 | No |
| 175 | TUBB6 |  |  | 7396 | -0.180 | -0.4820 | No |
| 176 | TCEAL8 |  |  | 7456 | -0.187 | -0.4863 | No |
| 177 | HNRNPA3 |  |  | 7470 | -0.188 | -0.4853 | No |
| 178 | CENPN |  |  | 7528 | -0.194 | -0.4893 | No |
| 179 | CMC2 |  |  | 7539 | -0.196 | -0.4879 | No |
| 180 | TPX2 |  |  | 7549 | -0.197 | -0.4863 | No |
| 181 | UBE2T |  |  | 7561 | -0.199 | -0.4849 | No |
| 182 | CDC42EP3 |  |  | 7562 | -0.199 | -0.4823 | No |
| 183 | BEX4 |  |  | 7566 | -0.199 | -0.4800 | No |
| 184 | SAE1 |  |  | 7570 | -0.200 | -0.4777 | No |
| 185 | BIRC5 |  |  | 7596 | -0.204 | -0.4779 | No |
| 186 | DCXR |  |  | 7713 | -0.220 | -0.4883 | No |
| 187 | EIF5 |  |  | 7763 | -0.227 | -0.4909 | No |
| 188 | RAD21 |  |  | 7785 | -0.231 | -0.4903 | No |
| 189 | MZT1 |  |  | 7838 | -0.242 | -0.4931 | Yes |
| 190 | TUBG1 |  |  | 7846 | -0.243 | -0.4907 | Yes |
| 191 | PDLIM7 |  |  | 7864 | -0.247 | -0.4894 | Yes |
| 192 | RBP1 |  |  | 7877 | -0.250 | -0.4874 | Yes |
| 193 | TIMM10 |  |  | 7925 | -0.261 | -0.4894 | Yes |
| 194 | SOX4 |  |  | 7958 | -0.268 | -0.4895 | Yes |
| 195 | H2AFX |  |  | 7965 | -0.269 | -0.4866 | Yes |
| 196 | STIP1 |  |  | 7971 | -0.270 | -0.4836 | Yes |
| 197 | CKS2 |  |  | 7972 | -0.270 | -0.4801 | Yes |
| 198 | HMGB2 |  |  | 7973 | -0.271 | -0.4765 | Yes |
| 199 | FAM162A |  |  | 7983 | -0.272 | -0.4739 | Yes |
| 200 | MAD2L2 |  |  | 7998 | -0.275 | -0.4719 | Yes |
| 201 | PPP1CC |  |  | 8035 | -0.282 | -0.4723 | Yes |
| 202 | MAP3K7CL |  |  | 8063 | -0.288 | -0.4716 | Yes |
| 203 | DTYMK |  |  | 8075 | -0.292 | -0.4690 | Yes |
| 204 | CDKN3 |  |  | 8079 | -0.293 | -0.4655 | Yes |
| 205 | CDKN2C |  |  | 8091 | -0.295 | -0.4628 | Yes |
| 206 | PHF19 |  |  | 8093 | -0.295 | -0.4590 | Yes |
| 207 | NUDCD2 |  |  | 8119 | -0.300 | -0.4579 | Yes |
| 208 | CCT5 |  |  | 8125 | -0.302 | -0.4545 | Yes |
| 209 | TCP1 |  |  | 8137 | -0.306 | -0.4517 | Yes |
| 210 | TMEM106C |  |  | 8146 | -0.308 | -0.4486 | Yes |
| 211 | VDAC3 |  |  | 8151 | -0.309 | -0.4449 | Yes |
| 212 | SAT1 |  |  | 8162 | -0.314 | -0.4419 | Yes |
| 213 | MORF4L2 |  |  | 8168 | -0.317 | -0.4383 | Yes |
| 214 | SRSF3 |  |  | 8191 | -0.323 | -0.4366 | Yes |
| 215 | HMGN1 |  |  | 8192 | -0.324 | -0.4323 | Yes |
| 216 | ILF2 |  |  | 8220 | -0.331 | -0.4310 | Yes |
| 217 | CBX1 |  |  | 8222 | -0.331 | -0.4267 | Yes |
| 218 | KIF5B |  |  | 8235 | -0.334 | -0.4237 | Yes |
| 219 | CKS1B |  |  | 8241 | -0.336 | -0.4198 | Yes |
| 220 | LSM5 |  |  | 8242 | -0.337 | -0.4154 | Yes |
| 221 | RBM8A |  |  | 8253 | -0.340 | -0.4120 | Yes |
| 222 | CCDC167 |  |  | 8260 | -0.342 | -0.4082 | Yes |
| 223 | PSMC3 |  |  | 8261 | -0.342 | -0.4036 | Yes |
| 224 | LDHA |  |  | 8263 | -0.343 | -0.3992 | Yes |
| 225 | SSNA1 |  |  | 8276 | -0.347 | -0.3960 | Yes |
| 226 | UBE2C |  |  | 8278 | -0.348 | -0.3915 | Yes |
| 227 | TUBB4B |  |  | 8356 | -0.371 | -0.3955 | Yes |
| 228 | NUDT1 |  |  | 8359 | -0.373 | -0.3908 | Yes |
| 229 | CLIC1 |  |  | 8368 | -0.378 | -0.3867 | Yes |
| 230 | PLIN3 |  |  | 8403 | -0.388 | -0.3854 | Yes |
| 231 | HMGB3 |  |  | 8427 | -0.399 | -0.3828 | Yes |
| 232 | PTTG1 |  |  | 8446 | -0.406 | -0.3795 | Yes |
| 233 | LMNA |  |  | 8478 | -0.423 | -0.3775 | Yes |
| 234 | CACYBP |  |  | 8486 | -0.428 | -0.3726 | Yes |
| 235 | SNRNP25 |  |  | 8494 | -0.432 | -0.3677 | Yes |
| 236 | NUCKS1 |  |  | 8502 | -0.435 | -0.3627 | Yes |
| 237 | CALM3 |  |  | 8517 | -0.443 | -0.3585 | Yes |
| 238 | MRPL51 |  |  | 8522 | -0.445 | -0.3531 | Yes |
| 239 | SNRPD1 |  |  | 8537 | -0.451 | -0.3487 | Yes |
| 240 | C12orf75 |  |  | 8546 | -0.456 | -0.3436 | Yes |
| 241 | GLIPR2 |  |  | 8547 | -0.457 | -0.3375 | Yes |
| 242 | RAN |  |  | 8555 | -0.463 | -0.3322 | Yes |
| 243 | HNRNPA1 |  |  | 8570 | -0.469 | -0.3276 | Yes |
| 244 | RPA3 |  |  | 8572 | -0.471 | -0.3215 | Yes |
| 245 | PRDX1 |  |  | 8584 | -0.476 | -0.3165 | Yes |
| 246 | PTGES3 |  |  | 8587 | -0.479 | -0.3104 | Yes |
| 247 | RAB13 |  |  | 8590 | -0.481 | -0.3042 | Yes |
| 248 | RPL31 |  |  | 8592 | -0.482 | -0.2980 | Yes |
| 249 | HMGN2 |  |  | 8608 | -0.496 | -0.2931 | Yes |
| 250 | PDLIM4 |  |  | 8630 | -0.512 | -0.2888 | Yes |
| 251 | METRN |  |  | 8638 | -0.518 | -0.2827 | Yes |
| 252 | CYCS |  |  | 8640 | -0.519 | -0.2760 | Yes |
| 253 | HMGB1 |  |  | 8653 | -0.527 | -0.2704 | Yes |
| 254 | EEF2 |  |  | 8670 | -0.540 | -0.2651 | Yes |
| 255 | HMGA1 |  |  | 8675 | -0.543 | -0.2583 | Yes |
| 256 | CDKN1A |  |  | 8679 | -0.545 | -0.2515 | Yes |
| 257 | ANP32B |  |  | 8680 | -0.545 | -0.2442 | Yes |
| 258 | SLC25A5 |  |  | 8681 | -0.546 | -0.2370 | Yes |
| 259 | SNRPB |  |  | 8683 | -0.547 | -0.2299 | Yes |
| 260 | GLRX5 |  |  | 8685 | -0.548 | -0.2228 | Yes |
| 261 | H2AFV |  |  | 8693 | -0.554 | -0.2162 | Yes |
| 262 | RHEB |  |  | 8694 | -0.554 | -0.2089 | Yes |
| 263 | C4orf3 |  |  | 8705 | -0.565 | -0.2026 | Yes |
| 264 | MT2A |  |  | 8740 | -0.598 | -0.1986 | Yes |
| 265 | CALM2 |  |  | 8744 | -0.600 | -0.1910 | Yes |
| 266 | TUBB |  |  | 8763 | -0.621 | -0.1848 | Yes |
| 267 | DEK |  |  | 8770 | -0.627 | -0.1772 | Yes |
| 268 | COMMD6 |  |  | 8772 | -0.628 | -0.1690 | Yes |
| 269 | H2AFZ |  |  | 8774 | -0.630 | -0.1608 | Yes |
| 270 | STMN1 |  |  | 8783 | -0.638 | -0.1533 | Yes |
| 271 | LSM3 |  |  | 8803 | -0.655 | -0.1468 | Yes |
| 272 | FABP7 |  |  | 8811 | -0.661 | -0.1388 | Yes |
| 273 | RANBP1 |  |  | 8819 | -0.669 | -0.1308 | Yes |
| 274 | BEX1 |  |  | 8833 | -0.689 | -0.1232 | Yes |
| 275 | H3F3B |  |  | 8846 | -0.705 | -0.1152 | Yes |
| 276 | LSM4 |  |  | 8853 | -0.712 | -0.1065 | Yes |
| 277 | RPL34 |  |  | 8877 | -0.744 | -0.0993 | Yes |
| 278 | PTMA |  |  | 8894 | -0.764 | -0.0910 | Yes |
| 279 | CHCHD2 |  |  | 8898 | -0.770 | -0.0812 | Yes |
| 280 | UBE2S |  |  | 8901 | -0.772 | -0.0712 | Yes |
| 281 | S100B |  |  | 8908 | -0.784 | -0.0615 | Yes |
| 282 | OST4 |  |  | 8925 | -0.829 | -0.0524 | Yes |
| 283 | EEF1A1 |  |  | 8958 | -0.915 | -0.0439 | Yes |
| 284 | TUBA1B |  |  | 8980 | -1.103 | -0.0318 | Yes |
| 285 | FTH1 |  |  | 8981 | -1.131 | -0.0168 | Yes |
| 286 | FTL |  |  | 8984 | -1.286 | 0.0000 | Yes |
Table: GSEA details [plain text format]

  

Fig 2: FELDMAN\_2020\_G2-M: Random ES distribution      
 Gene set null distribution of ES for **FELDMAN\_2020\_G2-M**
